# Acetylation dynamics of MrATG4 governing autophagy-mediated conidiation in an entomopathogenic fungus

**DOI:** 10.1371/journal.ppat.1013883

**Published:** 2026-01-20

**Authors:** Deshui Yu, Yulong Wang, Rui Xie, Rong Zhou, Zhenbang Liu, Najie Shi, Xiangyun Xie, Yang Yang, Jiaojiao Qu, Guang Yang, Bo Huang

**Affiliations:** 1 Anhui Provincial Key Laboratory of Microbial Pest Control, Anhui Agricultural University, Hefei, China; 2 Hefei City Forestry Protection Center, Hefei, China; 3 Sericulture Research Institute, Anhui Academy of Agricultural Sciences, Hefei, China; 4 School of Life Sciences, University of Science and Technology of China, Hefei, China; 5 College of Agriculture and Biology, Liaocheng University, Liaocheng, China; 6 Biomass Molecular Engineering Center and Department of Materials Science and Engineering, Anhui Agricultural University, Hefei, Anhui, China; Chinese Academy of Sciences, CHINA

## Abstract

Conidial production is a critical factor determining the efficacy of entomopathogenic fungi as biocontrol agents. Autophagy, a fundamental cellular degradation process, plays an essential role in regulating fungal conidiation. However, the modulation of autophagy through acetylation, particularly concerning the autophagy-related protein ATG4, remains poorly understood in fungi. Here, we investigate the roles of the deacetylase MrSIR2–3 and the acetyltransferase MrKAT1 in *Metarhizium robertsii*, focusing on their impacts on autophagy and conidiation. Our findings demonstrate that deletion of MrSIR2–3 (Δ*Mrsir2–3*) leads to elevated autophagy levels, whereas loss of MrKAT1 (Δ*Mrkat1*) suppresses autophagy initiation; both alterations consequently impair conidiation. Interaction assays further reveal that the key autophagy factor MrATG4 is regulated by opposing acetylation and deacetylation mediated by MrKAT1 and MrSIR2–3, potentially via modification of lysine residues K69 and/or K77. This dynamic acetylation balance is essential for maintaining autophagy homeostasis and ensuring efficient conidiation. Collectively, our results provide novel insights into how the acetylation of ATG4 modulates autophagy, advancing our understanding of conidiation regulation in entomopathogenic fungi and highlighting potential targets for enhancing fungal biocontrol efficacy.

## Author summary

Autophagy is a vital cellular process that supports growth and reproduction in fungi by forming autophagosomes to digest and recycle cellular components. In this study, we explore the role of the autophagy-related protein MrATG4 in the autophagy and conidiation of the entomopathogenic fungus *Metarhizium robertsii*, a widely used biopesticide for natural pest control. We focus on the regulatory roles of MrSIR2–3, a deacetylase, and MrKAT1, an acetyltransferase, in modulating the acetylation status of MrATG4. Our findings reveal that deletion of MrSIR2–3 enhances autophagy, whereas deletion of MrKAT1 suppresses it, both significantly impacting conidia production. This study provides the first evidence that acetylation of ATG4 is crucial for autophagy and conidia formation in fungi. These insights deepen our understanding of fungal growth and reproductive regulation and offer potential strategies for optimizing biological pest control.

## Introduction

Autophagy is a highly conserved cellular degradation pathway essential for maintaining cellular homeostasis by facilitating the turnover and reutilization of proteins and organelles, particularly under stress conditions such as nutrient starvation or pathogen infection [1,2]. This process involves the formation of autophagosomes that sequester cytoplasmic content and deliver it to the lysosomal compartment for degradation [3]. In filamentous fungi, autophagy is pivotal for various aspects of fungal biology, including growth, differentiation, conidiation, and pathogenesis [4–6].

The molecular underpinnings of autophagy have been extensively elucidated through the study of autophagy-related (ATG) genes, beginning with the identification of ATG1 in *Saccharomyces cerevisia*e [3,7]. *Metarhizium robertsii* serves as both an entomopathogenic fungus and a plant symbiont, with at least ten *Mratg* genes playing essential roles in nutrient utilization, underscoring the critical importance of autophagy [4,8–11]. Among these, ATG4, a cysteine protease, is indispensable for the processing of ATG8, a ubiquitin-like protein essential for autophagosome formation and autophagic flux [3,8]. Functional studies in fungi such as *Magnaporthe oryzae* and *Aspergillus oryzae* have demonstrated that ATG4 deletion results in defects in both autophagy and conidiation, underscoring its vital role in fungal development and virulence [5,6].

The regulation of autophagy is intricately controlled by post-translational modifications (PTMs) of ATG proteins, with acetylation emerging as a critical PTM influencing this process [2,12–15]. Acetylation modulates the activity, stability, and interactions of key autophagy regulators, thereby fine-tuning autophagic responses [14]. Acetyltransferases, such as CBP, P300, and TIP60, and deacetylases, including HDAC6 and the NAD ⁺ -dependent deacetylase SIRT1, have been extensively characterized for their roles in autophagy regulation [14]. For example, acetylation of key autophagy factors such as ATG5, ATG7, ATG8, and ATG12 by P300 promotes autophagy, while deacetylation by SIRT1 directly regulates these proteins to modulate autophagic activity [16,17].

Recent studies in mammalian systems have identified acetylation of ATG4B as a pivotal mechanism regulating the initiation of autophagy during starvation. This acetylation-dependent modulation of ATG4B activity underscores the conserved role of acetylation in autophagy regulation across eukaryotes [18]. However, the role of ATG4 acetylation in fungi remains unexplored, presenting a significant gap in our understanding of autophagy regulation in these organisms.

In fungal systems, acetylation of ATG-like genes is essential for maintaining autophagy homeostasis, which in turn influences fungal growth, conidiation, and virulence. For instance, in *Magnaporthe oryzae*, the acetyltransferase Gcn5 acetylates ATG7, thereby affecting starvation-induced autophagy and phototropism [19]. Similarly, in *Fusarium graminearum*, Gcn5-mediated acetylation of ATG8 induces autophagy, impacting fungal growth, competitive fitness, and virulence [20]. Additionally, in *F. oxysporum*, the deacetylation of FolGsk3K271 by the deacetylase FolSIR2 modulates fungal pathogenicity [21].

Despite these advancements, the acetylation of ATG4 and its regulatory implications in fungal autophagy remain unexplored. Given the central role of ATG4 in autophagosome formation and autophagic flux [1,2], understanding its regulation through acetylation could unveil novel mechanisms critical for autophagy-mediated processes such as conidiation and pathogenicity. Elucidating the acetylation dynamics of ATG4 in entomopathogenic fungi like *M. robertsii* is particularly pertinent [22–26], as efficient conidial production is a bottleneck in the practical application of fungi-based biopesticides, fertilizers, and plant immunity promoters [9,11].

This study aimed to investigate the acetylation regulation of MrATG4 by the deacetylase MrSIR2–3 and the acetyltransferase MrKAT1 in *M. robertsii*. By systematically analyzing mutants of the SIR2 family and identifying the specific lysine residues targeted for acetylation, we seek to elucidate the molecular mechanisms by which acetylation modulates autophagy and conidiation. Our findings provide novel insights into the post-translational regulation of autophagy in entomopathogenic fungi, offering potential targets for enhancing fungal biocontrol efficacy.

## Results

### Bioinformatic analysis and subcellular localization of SIR2s homologs

**Fig 1:**
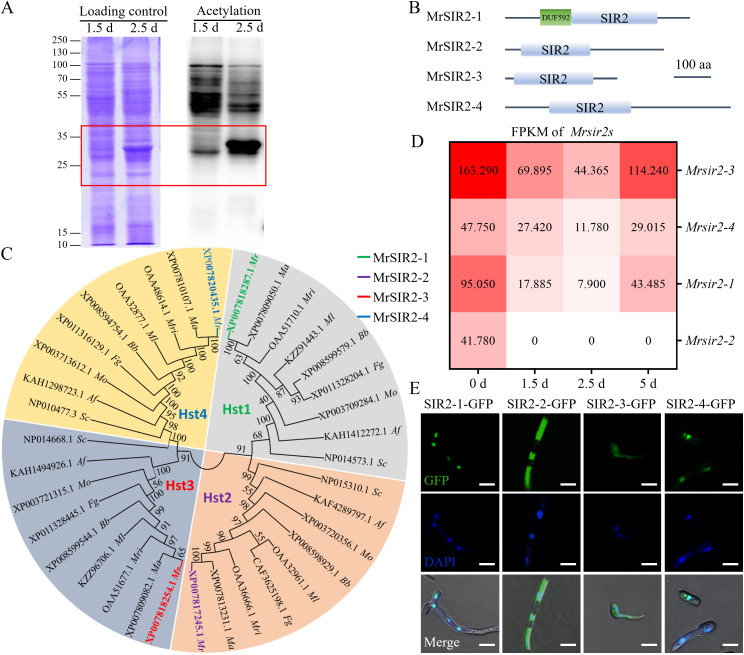
Acetylation and SIR2 proteins in *M. robertsii.* **(A)** Immunoblot analysis was performed to assess lysine acylation in mycelial growth and the initial conidiation stages. Coomassie blue staining of the gel served as the loading control, with 20 µg of protein loaded in each lane. **(B)** Schematic representation illustrating four *M. robertsii* SIR2 proteins. DUF592, unknown function domain of the protein; SIR2, silent information regulator 2; aa, amino acids. **(C)** Alignment of *M. robertsii* and other fungal SIR2 proteins. Protein sequences were aligned, and the phylogenetic tree was generated using MEGA11. *Mr*: *M. robertsii*; *Ma*: *M. acridum*; *Mri*: *M. rileyi*; *Ml*: *Moelleriella libera*; *Bb*: *Beauveria bassiana*; *Fg*: *Fusarium graminearum*; *Mo*: *Magnaporthe oryzae*; *Af*: *Aspergillus fumigatus*; *Sc*: *Saccharomyces cerevisiae*. **(D)** Quantification of *Mrsir2* genes expression in *M. robertsii* using RNA-seq at different developmental stages. **(E)** Subcellular localization of MrSIR2 proteins. Scale bar = 5 μm.

To investigate the role of lysine acetylation (Kac) in fungal development, we initially examined mycelial growth and the early stages of conidiation in *M. robertsii*. Western blot analysis revealed a significant increase in global Kac levels during initial conidiation compared to mycelial growth, with prominent bands observed between 25–35 kDa, which suggests that acetylation plays a regulatory role in conidiation (Fig 1A).

The SIR2 family, recognized as NAD ^+^ -dependent deacetylases, is widely reported to be involved in various biological processes. However, its role in entomopathogenic fungi remains poorly understood. Orthologs of the four SIR2 proteins (HST1, HST2, HST3, HST4) from *S. cerevisiae* were identified in the *M. robertsii* genome: MrSIR2–1 (MAA_02098), MrSIR2–2 (MAA_01056), MrSIR2–3 (MAA_02065), and MrSIR2–4 (MAA_04246) (Fig 1B). Domain architecture analysis confirmed the presence of NAD ^+^ -dependent protein/histone deacetylation domains within these homologs. Phylogenetic analysis further demonstrated that all MrSIR2s are highly conserved and cluster within the four subfamilies corresponding to HST1–4, respectively (Fig 1C).

Transcriptomic analysis via RNA-seq across different developmental stages—conidia (0 d), initial mycelial growth (1.5 d), initiation of conidiation (2.5 d), and conidial maturation (5 d)—revealed that *Mrsir2–3* exhibits significantly higher expression levels compared to other *Mrsir2s* during mycelial growth and conidiation (Fig 1D).

Subcellular localization analysis using GFP fusion constructs and DAPI staining revealed distinct subcellular distributions of the MrSIR2 proteins (Fig 1E). Specifically, MrSIR2–1-GFP and MrSIR2–4-GFP were predominantly localized to the nucleus, whereas MrSIR2–2-GFP and MrSIR2–3-GFP were primarily found in the cytoplasm. This distribution suggests distinct functional roles for the MrSIR2 homologs within different cellular compartments.

### The role of SIR2s in deacetylase activity and conidial production

To determine the functional roles of MrSIR2s, we generated targeted gene deletion mutants (Δ*Mrsir2–1*, Δ*Mrsir2–*2, Δ*Mrsir2–3*, Δ*Mrsir2–4*) and complemented strains through *Agrobacterium*-mediated homologous recombination (S1 Fig). PCR validation confirmed the successful generation of gene knockouts (S2 Fig).

**Fig 2:**
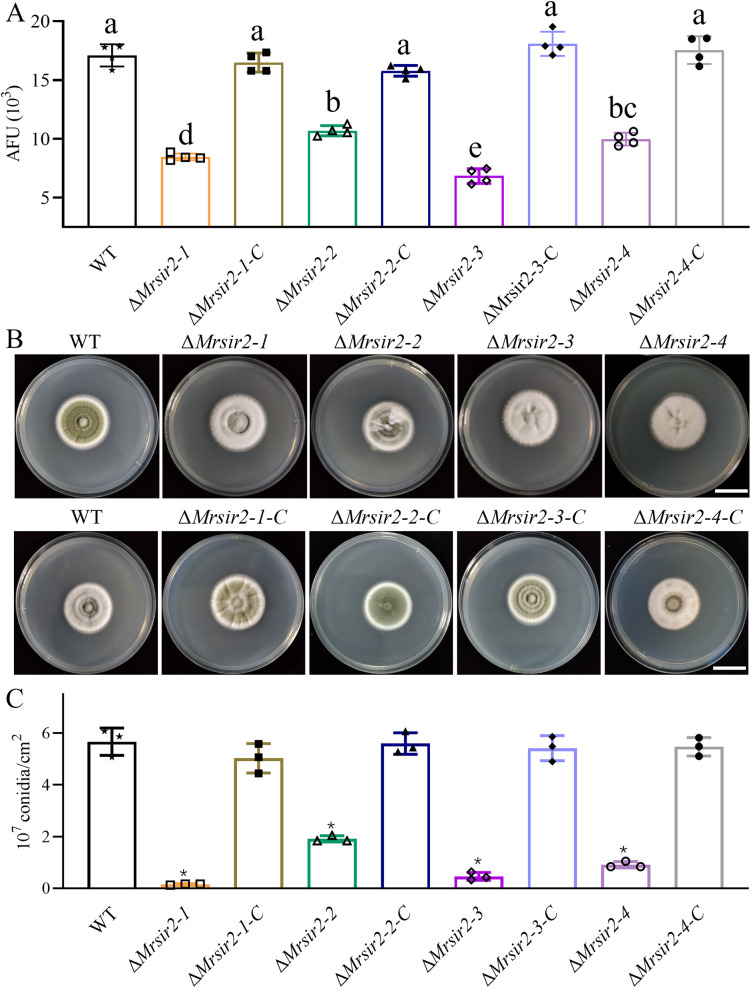
Contribution of MrSIR2s to conidial yields and deacetylase activity. **(A)** Intracellular deacetylase activity in different strains measured in arbitrary fluorescence units (AFU). * *p* < 0.01. **(B)** Colony phenotypes of WT and mutant strains on PDA media after 7 days of growth at 25 °C. **(C)** Quantification of conidial yields from different strains after two weeks of growth on PDA. * *p* < 0.01.

Deacetylase activity assays, performed using a fluorescent AMC substrate, revealed that all Δ*Mrsir2* mutant strains exhibited significantly reduced deacetylase activity compared to the wild-type strain (WT) (Fig 2A). Notably, the deacetylase activity of the Δ*Mrsir2–3* strain was lower than that of the other three mutant strains, indicating that MrSIR2–3 plays a crucial role in fungal deacetylation processes.

Phenotypic assays conducted on Potato Dextrose Agar (PDA) demonstrated that deletion of *Mrsir2* genes did not impact fungal growth diameters, suggesting that *Mrsir2* genes do not regulate overall mycelial growth under these conditions (Fig 2B). Assessments of conidial yields revealed substantial reductions in conidial production across all Δ*Mrsir2* mutants compared to the WT strain. Specifically, Deletion of *Mrsir2–1*, *Mrsir2–2*, *Mrsir2–3*, and *Mrsir2–4* exhibited reductions in conidial production by 97%, 66%, 92%, and 84%, respectively (Fig 2C). Consistent results were obtained from conidiation analyses performed on different media and at various time points, with *Mrsir2* mutants consistently showing significantly lower conidial production than the WT strain (S3 Fig). Complementation of these mutants restored conidiation to levels comparable to the WT strain, thereby confirming the essential roles of *Mrsir2* genes in conidiation. Transcriptomic analysis of conidiation-associated genes further indicated that deletion of *Mrsir2* genes leads to significant downregulation of key regulatory genes, such as *stc*, *hymA*, *flbC*, *phiA*, *fadA*, *flbD*, *sakA*, *abaA*, *brlA*, *stuA*, *medA*, and *wetA*, during conidiation (S4 Fig).

Collectively, these findings demonstrate that the MrSIR2 proteins exhibit functional deacetylase activity and play essential roles in efficient conidial production in *M. robertsii*.

### Deacetylase MrSIR2–3 deacetylates MrATG4

Given the elevated expression of *Mrsir2–3* during conidiation and its cytoplasmic localization, combined with its major role in deacetylation at the assayed stage and significant impact on conidial production, we prioritized *Mrsir2–3* for further investigation. Using yeast two-hybrid screening with pGBKT7-MrSIR2–3 as bait, we identified 24 potential interacting proteins based a genome-wide screening, including MrATG4, a key autophagy-related protein with a molecular weight of 28.4 kDa (S1 Table).

**Fig 3:**
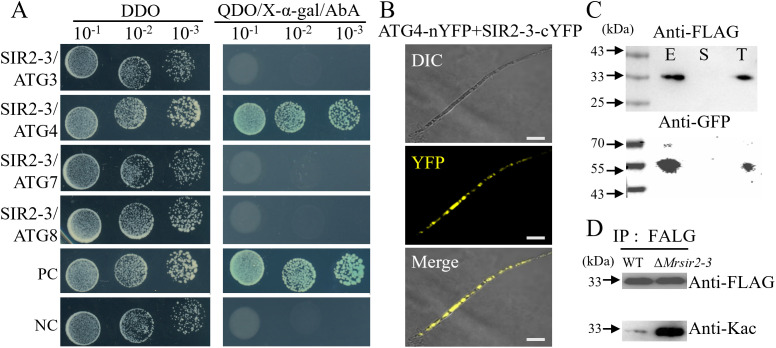
Interaction and deacetylation between MrSIR2-3 and MrATG4 in *M. robertsii.* **(A)** Yeast two-hybrid analysis demonstrating the interaction between MrSIR2-3 and the ATG8-conjugation system proteins. DDO, SD-T-L; QDO, SD-T-L-A-H. **(B)** Bimolecular fluorescence complementation (BiFC) assays for detecting in *vivo* protein interactions. Scale bar: 5 μm. **(C)** Co-immunoprecipitation assays. Total proteins, suspensions, and proteins eluted from anti-FLAG agarose from transformants co-expressing MrSIR2-3-GFP and MrATG4-FLAG. The blots were probed with anti-FLAG or anti-GFP antibodies. T, total; S, suspensions; E, elution. **(D)** FLAG co-IP and anti-acetyl-lysine immunoblot analysis of WT vs. Δ*Mrsir2-3* strains grown on PDA (2.5 d).

Further yeast two-hybrid assays revealed that MrSIR2–3 specifically interacts with MrATG4 but not with other components of the ATG8-conjugation system (Fig 3A). To validate this interaction, we conducted bimolecular fluorescence complementation (BiFC) and co-immunoprecipitation (co-IP) assays. Both methods confirmed the physical association between MrSIR2–3 and MrATG4 (Figs 3B, 3C, S5 and S6).

To assess the levels of MrATG4 acetylation, we constructed and harvested MrATG4-FLAG strains of the WT and Δ*Mrsir2–3* mutant at early conidiation (2.5 days). FLAG immunoprecipitates were immunoblotted with an anti-pan-acetylated lysine antibody, revealing higher MrATG4 acetylation levels in the WT strain compared to the Δ*Mrsir2–3* mutant (Fig 3D). These findings indicate that MrSIR2–3 directly interacts with and deacetylates MrATG4, thereby modulating its acetylation state during conidiation.

### Regulation of autophagy by MrSIR2–3 and MrATG4 during conidiation

**Fig 4:**
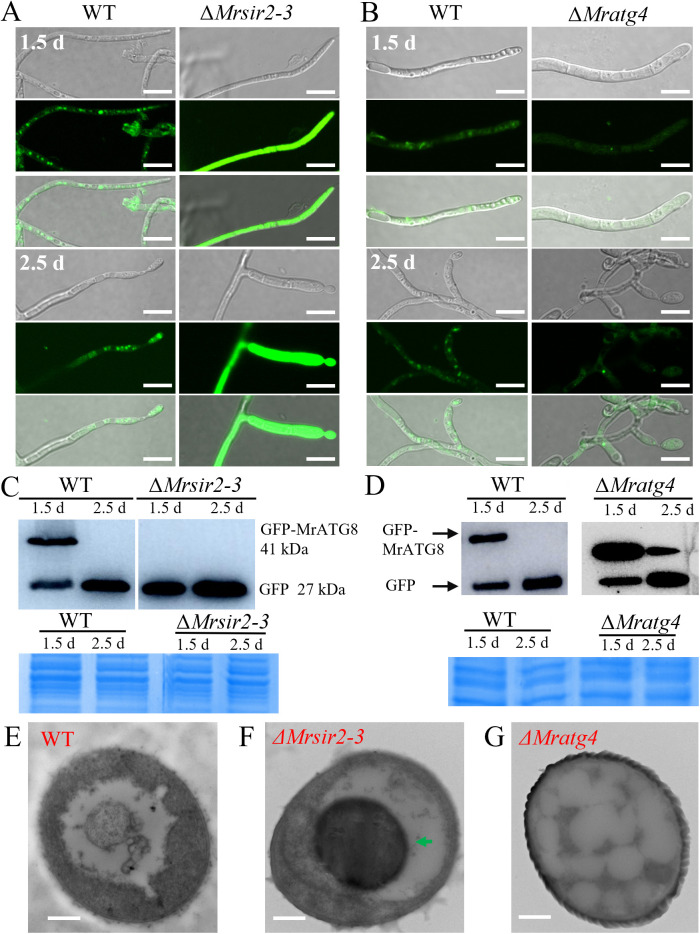
Critical roles of *Mrsir2-3* and *Mratg4* in autophagy of *M. robertsii.* **(A** **and** **B)** Cellular localization of autophagosomes during the initial mycelial growth (1.5 d) and initial conidia production stages (2.5 d). Scale bar: 10 μm. **(C** **and** **D)** Analysis of GFP-MrATG8 degradation in the WT and mutant mycelial cells. Immunoblotting was performed with anti-GFP. For each analysis, parallel protein gels were stained as references. **(E - G)** Transmission Electron Microscopy (TEM) analysis of the vacuolar localization features of Autophagosomes in the mycelial structures during conidiation in both wild-type WT and mutant strains. Autophagic bodies are indicated by arrows in the Δ*Mrsir2-3* cell. Bar: 2 μm.

To elucidate the roles of MrSIR2–3 and MrATG4 in autophagy regulation, we utilized a GFP-MrATG8 autophagy marker and introduced it into WT, Δ*Mrsir2–3*, and Δ*Mratg4* mutant strains. In the WT, autophagosomes and GFP-MrATG8 fluorescence were readily observed during the initial stages of conidiation (Fig 4A and 4B). In contrast, the Δ*Mrsir2–3* mutant exhibited an increased accumulation of autophagosomes and heightened GFP-MrATG8 fluorescence within the cytoplasm, indicative of elevated autophagic activity (Figs 4A–4D, S7 and S8). Conversely, the Δ*Mratg4* strain displayed minimal autophagosomes and faint GFP-MrATG8 fluorescence, suggesting impaired autophagy (Fig 4E and 4F). Transmission electron microscopy (TEM) further corroborated these observations, showing an abundance of autophagic bodies within vacuoles of the Δ*Mrsir2–3* mutant and a marked absence of autophagic bodies in Δ*Mratg4* (Fig 4G).

These results demonstrate that MrSIR2–3 acts as a negative regulator of autophagy, likely by deacetylating MrATG4, which is essential for proper autophagic function during conidiation.

### Acetyltransferase MrKAT1 interacts with MrATG4

**Fig 5:**
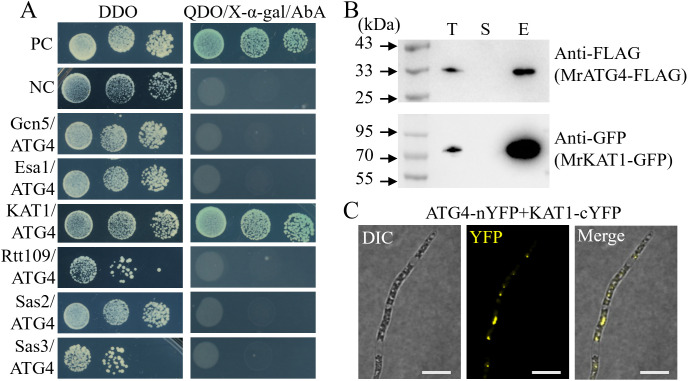
MrKAT1 interacted with MrATG4. **(A)** Yeast two-hybrid analysis of the interaction between MrATG4 and the Nε-lysine acetyltransferase proteins. DDO, SD-T-L; QDO, SD-T-L-A-H. **(B)** Co-IP assays. Western blots of total proteins, suspensions and proteins eluted from anti-FLAG agarose from transformants co-transformed MrKAT1-GFP and MrATG4-FLAG, MrATG4-FLAG and MrKAT1-GFP were detected with anti-FLAG or anti-GFP antibodies. T, total; S, suspensions; E, elution. **(C)** BiFC assays for detecting *in vivo* protein interactions. Scale bar: 5 μm.

To identify potential acetyltransferases that interact with MrATG4, we performed yeast two-hybrid assays using a panel of acetyltransferase homologs from *S. cerevisiae* (Gcn5 (MAA_05172), Esa1 (MAA_02727), Hat1 (MAA_05971), Rtt109 (MAA_01374), Sas2 (MAA_04679), and Sas3 (MAA_02282) as bait. This screen identified the Hat1 homolog MAA_05971 as a specific interacting partner of MrATG4 (Fig 5A). We therefore designated this protein MrKAT1. To validate this interaction, we conducted co-IP assays. Co-expression of MrATG4-FLAG and MrKAT1-GFP in the wild-type (WT) strain resulted in the co-precipitation of MrKAT1 with MrATG4 using anti-FLAG beads, confirming their physical association (Fig 5B). Additionally, bimolecular fluorescence complementation (BiFC) assays were performed to confirm the *in vivo* interaction between MrKAT1 and MrATG4. The presence of YFP fluorescence indicated that MrKAT1 and MrATG4 interact in living cells (Figs 5C, S5 and S6).

### Role of *Mrkat1* in autophagy and conidiation

**Fig 6:**
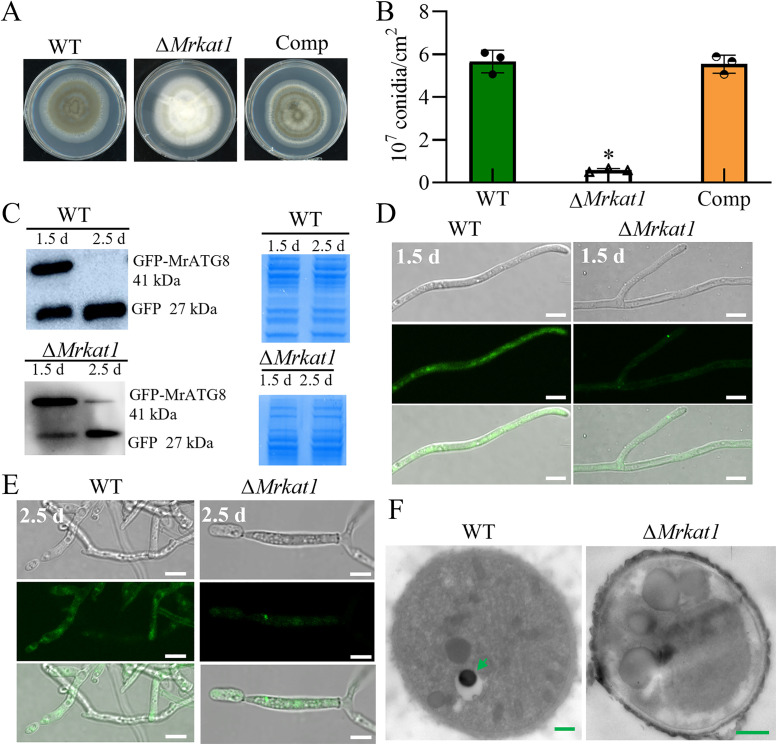
Contribution of MrKAT1 to autophagy and conidial production in *M. robertsii.* **(A and B)** Colony phenotyping of WT and mutant strains on PDA media after 14 days of growth at 25 °C. * *p* < 0.01. **(C)** Analysis of GFP-MrATG8 degradation in WT and mutant mycelial cells. Immunoblotting was performed with anti-GFP. For each analysis, the parallel-running protein gels were stained as references. * *p* < 0.01. **( D and E)** Cellular location of Autophagosomes during the initial conidia production (2.5 d) and initial mycelial growth (1.5 d) stages. Scale bar: 5 μm. **(F)** TEM Analysis: This image illustrates the vacuolar localization features of Autophagosomes in the mycelial structures during the conidiation phase for both WT and Δ*Mrkat1* strains. The autophagic bodies are arrowed in the WT cell. Bar: 1 μm.

*Mrkat1* deletion mutant and a complemented strain (Δ*Mrkat1-C*) were generated to investigate the role of MrKAT1 in autophagy and conidiation (Figs 6 and S9). Phenotypic analysis revealed that the Δ*Mrkat1* mutant exhibited a significant reduction in conidial production compared to WT and Δ*Mrkat1-C* (Fig 6A and B). Autophagic flux assessments using GFP-MrATG8 indicated that the Δ*Mrkat1* mutant displayed reduced autophagy levels, similar to the Δ*Mratg4* mutant (Fig C–E and S9). Immunoblotting further quantified autophagic flux by assessing the ratio of free GFP to total GFP-MrATG8, revealing a marked decrease in free GFP in Δ*Mrkat1* (Fig C–E). TEM analysis confirmed the reduced presence of autophagic bodies in Δ*Mrkat1*, akin to Δ*Mratg4* (Fig 6F).

These findings establish MrKAT1 as a positive regulator of autophagy, functioning antagonistically to MrSIR2–3, and essential for efficient conidiation in *M. robertsii*.

### Acetylation enhances ATG4 proteolytic activity

**Fig 7:**
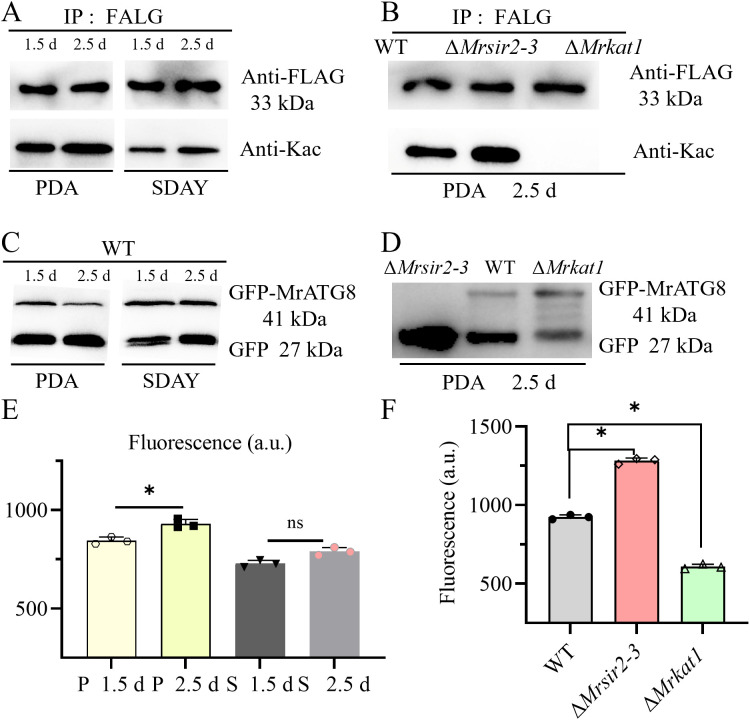
The connection between ATG4 acetylation and ATG4 activity. **(A)** The WT strain was cultured on PDA or SDAY for 1.5 d, 2.5 d and proteins were immunoprecipitated with antibody to FLAG followed by immunoblotting with antibody to acetylated-lysine. **(B)** Western blot analyses of the acetylation levels (pan anti-Kac, lower panel) and amount (anti-FLAG, upper panel) of MrATG4-FLAG protein in WT, Δ*Mrsir2-3* and Δ*Mrkat1* strains. Proteins were immunoprecipitated with anti-FLAG antibody agarose beads and analyzed using the indicated antibodies. **(C)** GFP-MrATG8 degradation in WT cells cultured on PDA or SDAY for 1.5 d and 2.5 d was analyzed by immunoblotting using an anti-GFP antibody. For each analysis, the parallel-running protein gels were stained as references (S11 Fig). **(D)** Analysis of GFP-MrATG8 degradation in WT and mutant cells cultured on PDA for 2.5 d was performed by anti-GFP immunoblotting. For each experiment, duplicate protein gels were run in parallel and stained as loading controls (S11 Fig). (**E and F**) After treatment as in (C or D), lysates from strains grown under different conditions were assayed for ATG4 activity using the fluorogenic substrate AU4S.

ATG4 acetylation and enzyme activity were significantly higher in fungi grown on PDA compared to those grown on SDAY at both 1.5 days and 2.5 days of cultivation (Figs 7A, 7B, S10 and S11). Specifically, ATG4 acetylation and enzyme activity in fungi grown on both PDA and SDAY were elevated at 2.5 days (the stage of early conidiation) compared to those at 1.5 days. Moreover, MrATG4 acetylation levels were markedly increased in the Δ*Mrsir2–3*, whereas they were nearly abolished in the Δ*Mrkat1* (Fig 7B). Autophagy levels, monitored by ATG8 cleavage, were found to be correlated with the acetylation status of MrATG4 (Figs 7C–7F and S11). Specifically, autophagy levels in the WT were higher at 2.5 days of cultivation compared to 1.5 days. Compare to the WT, autophagy levels of Δ*Mrkat1* were significantly reduced while substantially increased in the Δ*Mrsir2–3*. These experiments demonstrated that dynamic acetylation of MrATG4, modulated during fungal asexual reproduction, regulates autophagy to facilitate conidiation.

### Acetylation of MrATG4 at residues K69 and K77 governs autophagy and conidiation

**Fig 8:**
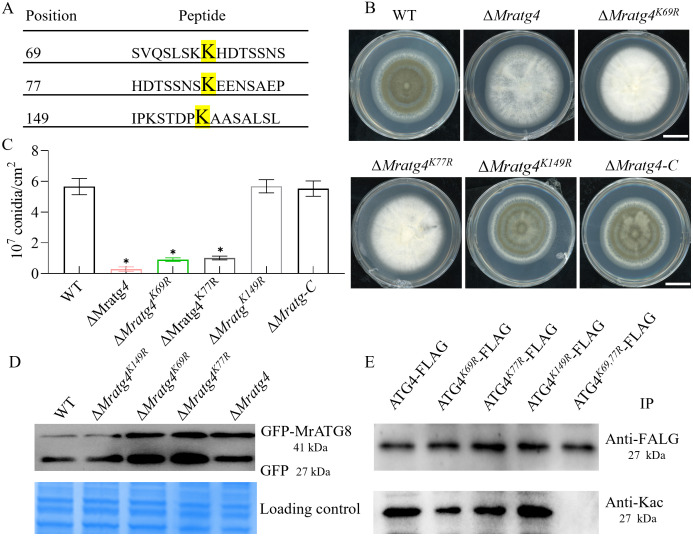
Regulation of autophagy and conidiation by MrATG4 acetylation. **(A)** Prediction of acetylation sites of MrATG4. Bioinformatics forecast through the prediction website PAIL identified three potential acetylation sites. **(B** **and** **C)** Colony phenotyping of WT and mutant strains on PDA media after 7 days of growth at 25 °C. Quantification of conidial yields by different strains after growth on PDA for 7 days. * *p* < 0.01. **(D)** Analysis of GFP-MrATG8 degradation in WT and mutant mycelial cells. Immunoblotting was performed with anti-GFP. For each analysis, the parallel-running protein gels were stained as references. **(E)** IP assays. Transformants expressing FLAG‑fused MrATG4 and its mutants, including MrATG4^*K69R*^-FLAG, MrATG4^*K77R*^-FLAG, MrATG4^*K149R*^-FLAG, MrATG4^*K69,77R*^-FLAG, were cultured on PDA medium for 2.5 days. Proteins were then eluted using anti‑FLAG agarose and analyzed by immunoblotting with an Kac antibody.

Given the interactions between MrATG4, MrSIR2–3, and MrKAT1, we hypothesized that MrATG4 is subject to acetylation and deacetylation, modulating its activity in autophagy regulation. Bioinformatics analysis predicted three potential acetylation sites on MrATG4 (K69, K77, K149) (Fig 8A). A triple lysine-to-arginine mutant (3K-R) and three single-site mutants (K69R, K77R, K149R) via site-directed mutagenesis were generated, respectively. Conidial production assays revealed that the *Mratg4*, K69R and K77R mutants exhibited significantly impaired conidiation compared to WT, while the K149R mutant did not show significant defects (Figs 8B, 8C and S12). Autophagic flux analysis using GFP-MrATG8 corroborated these findings, showing disrupted autophagy in K69R and K77R mutants (Fig 8D). In addition, strains expressing FLAG-tagged MrATG4 (MrATG4-FLAG) and its point mutant strains (ATG4^*K69R*^-FLAG, ATG4^*K77R*^-FLAG, ATG4*^K^*^*149R*^-FLAG, ATG4^*K69,7R7*^-FLAG) were constructed, and the corresponding proteins were purified using anti-FLAG magnetic beads. Immunoblotting revealed that acetylation levels (detected by an anti-acetyl-lysine antibody) were substantially reduced in the K69R and K77R single mutants, and completely abolished in the K69/77R double mutant. These results demonstrate that lysine residues K69 and K77 of MrATG4 are crucial for its function in autophagy regulation and conidiation. Additionally, MrSIR2–3 and MrKAT1 are potentially associated with these residues, suggesting a possible regulatory interaction and ATG4 may be acetylated at lysine residues 69 and 77.

## Discussions

Autophagy plays a pivotal role in the lifecycle of *M. robertsii*, particularly in the formation of conidia, which are essential for its efficacy as a biocontrol agent. This study elucidates the intricate regulation of autophagy through the dynamic acetylation and deacetylation of the autophagy-related protein ATG4 by the acetyltransferase MrKAT1 and the deacetylase MrSIR2–3, respectively.

**Fig 9:**
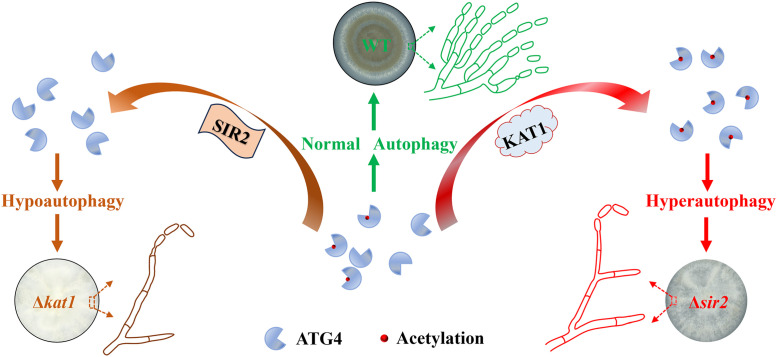
Schematic representation of the ATG4 acetylation regulating autophagy mediated conidiation in fungi. The homeostasis of ATG4 acetylation, mediated by SIR2/KAT1, regulates conidial production by influencing autophagy initiation. Acetyltransferase KAT1 enhances cysteine protease ATG4 activity, promoting hypo-autophagy during initial conidial production. Meanwhile, SIR2-mediated ATG4 deacetylation leads to fungal hyper-autophagy. Both hypo-autophagy and hyper-autophagy result in reduction in conidial production. The proper balance of autophagy ensures normal conidiation.

Our findings reveal that MrSIR2–3 acts as a negative regulator of autophagy by deacetylating MrATG4, thereby modulating its activity. Conversely, MrKAT1 promotes autophagy by acetylating MrATG4 possibly at specific lysine residues (K69 and K77). The balance between these opposing enzymatic activities ensures autophagy homeostasis, which is critical for efficient conidiation. Disruption of the balance through the deletion of either MrSIR2–3 or MrKAT1 impaired autophagic flux and conidial production, highlighting the delicate regulation required for optimal fungal development (Fig 9).

As previously reported, autophagy is closely associated with the conidiation process in filamentous fungi [27–30]. The SIRT2 enzyme modulates cellular metabolism and ROS stress responses, thereby impacting broader physiological processes, including aging, autophagy, and apoptosis [31–34]. The 90% reduction in conidia production observed in this study could be attributed to either a primary defect in autophagy or a secondary imbalance in metabolic/ROS homeostasis triggered by the absence of *Mrsir2–3*. Our current experiments indicate that *Metarhizium* begins to develop sporulation structures after approximately 2.5 days of growth on PDA, which coincides with a notable induction of autophagy. However, the precise conditions that trigger autophagy, and the mechanisms by which MrSIR2–3 and MrKAT1 are activated to initiate conidiation, remain unclear. Further experiments, such as measuring ATP/AMP and NAD ⁺ /NADH ratios or conducting rescue experiments with an ATP-generating carbon source, are necessary to determine whether the sporulation defect is specifically linked to autophagy or secondary metabolic/ROS stress. These additional studies will provide more insights into the complex regulatory networks governing conidiation and stress responses in *Metarhizium.*

ATG4, as a key role in the regulation of autophagy by cutting and recycling ATG8 [13], has a significant impact on fungal autophagy and conidiation in fungi [1,5,6,28]. In our work, Δ*Mratg4* induced the loss of autophagy and conidiation, aligning with earlier reports [4]. Furthermore, we discovered that MrATG4 interacted with MrSIR2–3 and MrKAT1, respectively. This interaction is in line with findings in mammals, where the deacetylation of ATG4B was established as a crucial step in initiating autophagy during starvation [18]. Our results on MrATG4 interaction and acetylation represent the first report in fungi, suggesting that ATG4 is regulated through multiple mechanisms and offering new insights into its acetylation control in fungi.

Interaction assays and IP assays suggest that MrATG4 may be regulated by MrKAT1 and MrSIR2–3, potentially through acetylation and deacetylation at lysine residues K69 and K77 (Figs 3, 5, 7 and 8). These findings enhance our understanding of post-translational modifications in autophagy regulation. These specific acetylation sites are critical for MrATG4's proteolytic activity, directly impacting ATG8 processing and autophagosome formation. In contrast, the K149R mutant showed no significant effects on MrATG4 function, indicating that not all predicted acetylation sites contribute equally to ATG4 function, highlighting the specificity of acetylation-mediated regulation.

While our findings establish MrSIR2–3 and MrKAT1 as key regulators of autophagy-mediated conidiation through dynamic acetylation of MrATG4, the upstream signals that activate these opposing enzymatic activities remain unresolved. We hypothesize that environmental cues (e.g., nutrient depletion, oxidative stress) or developmental checkpoints may trigger NAD^+^-dependent activation of MrSIR2–3 deacetylase activity, while acetyl-CoA availability could regulate MrKAT1 acetyltransferase function. The temporal coordination between these activities likely establishes a reversible acetylation switch that fine-tunes autophagy flux during the vegetative-to-reproductive transition.

Autophagy is essential for conidiation, and deficiencies in ATG1, ATG4, and ATG15 significantly reduce autophagy homeostasis and conidiation in filamentous fungi [4,6,35,36]. In eukaryotes, autophagy is enhanced by stresses or starvation, facilitating the degradation of increasing toxic and damaged components. The recycling of cell material is then utilized for nutrient remobilization [37]. The typical life cycle of filamentous fungi involves asexual conidiation when environmental factors limit the vegetative phase [38]. While our results do not directly demonstrate that autophagy is specifically involved in nutrient acquisition during the early stages of conidiation, they clearly indicate that autophagy is essential for maintaining cellular homeostasis, likely supporting the energy and biosynthetic demands of conidiation. Additionally, we reveal how fungi maintain autophagy homeostasis during the conidiation process through the regulation of ATG4 acetylation dynamics.

The study highlights the potential of targeting acetylation pathways to manipulate fungal conidiation and, by extension, its biocontrol efficacy. Modulating the activity of MrKAT1 or MrSIR2–3 could provide novel strategies to enhance or suppress autophagy, thereby influencing fungal development. This has significant implications for the development of more effective fungi-based biopesticides, offering avenues to optimize spore production. In a broader context, the conservation of acetylation-dependent regulation of ATG4 across eukaryotes suggests that similar mechanisms may be at play in other organisms, including plants and animals. Future studies could explore the evolutionary conservation of this regulatory system and its implications in various biological processes beyond fungal conidiation.

Overall, our study provides comprehensive insights into the molecular mechanisms governing autophagy-mediated conidiation in *M. robertsii*. Dynamic acetylation of MrATG4, modulated during fungal asexual reproduction, regulates autophagy to facilitate conidiation. Moreover, by elucidating the roles of MrKAT1 and MrSIR2–3 in modulating MrATG4 acetylation, we advance the understanding of post-translational regulation in fungal development and open new avenues for enhancing the application of entomopathogenic fungi in biological control.

## Materials and methods

### Fungal strains and culture conditions

The *M. robertsii* strain ARSEF 23 (ATCC no. MYA-3075) was cultured on Potato Dextrose Agar (PDA, BD Difco, BD 213300) for two weeks in the dark. The resulting conidial suspension from this culture served as the starting material for *Agrobacterium*-mediated transformation (ATMT). Mycelia and conidia were harvested from PDA plates at 1.5 and 2.5 days, respectively, to evaluate autophagic effects at different developmental stages of the fungus. These conditions were employed for subsequent experiments, including Laser Scanning Confocal Microscopy (LSCM, Zeiss LSM 880, Oberkochen, Germany) and immunoblotting.

### Bioinformatics analysis

Putative *Mrsir2* and *Mrkat1* genes were identified from generalist sequences through online BLAST analysis on NCBI. Protein sequences of MrSIR2 were aligned from different fungi including *Metarhizium robertsii*, *M. acridum*; *M. rileyi*; *Moelleriella libera*; *Beauveria bassiana*; *Fusarium graminearum*; *Magnaporthe oryzae*; *Aspergillus fumigatus*; *Saccharomyces cerevisiae*. The SMART program was used for structural comparisons, and phylogenetic analysis was conducted using the neighbor-joining method in MEGA11 [39].

### Genes deletion and phenotype assays

Agrobacterium-mediated homologous recombination was employed for the gene knockout of *Mrsir2s*, *Mratg4*, and *Mrkat1*, following our previous studies [39,40]. Specifically, pDHt-bar (conferring glufosinate resistance) was utilized for gene knockout, while pDHt-ben (conveying benomyl resistance) was used for generating the complementary strain (Comp). At least three mutants were obtained for each gene knockout, and one strain was randomly selected for testing. All primers used in this are listed in S2 Table.

Phenotype assays for different strains were conducted as follows: 1 μL aliquots of the conidial suspension (1 × 10^7^ conidia/mL) were centrally spotted onto PDA. Additionally, a conidium suspension of 100 μL was spread on PDA, SDAY, CM plates for conidial production statistics after 7 and 14 days [39].

### Deacetylase activity assays

The total protein from each strain, treated with a 100 nM HDAC inhibitor TSA, underwent incubation with a fluorescent AMC peptide (Ac-Arg-His-Lys-Lys^ac^) that was acetylated through Nε-acetylated lysine [41]. Subsequently, the deacetylation activity of the enzyme within the samples was assessed using a fluorescence microplate reader (Synergy HTX, BioTek, America).

### Transcriptomics analysis

To delineate the expression patterns of genes, samples from the WT strain were collected at various stages of fungal development. These stages included the induction of two-week-old conidia on PDA and tissues cultured on PDA for 1.5 days, 2.5 days, and 5 days, respectively. RNA-Seq analysis was conducted by the Beijing Genomics Institute (BGI, Shenzhen, China), employing the fragments per kilobase per million mapped reads (FPKM) method as previously described [42]. Additionally, the relative expression of conidiation-associated genes was evaluated after culturing on PDA for 2.5 days [39].

### Protein fusions and localization assays

To investigate the subcellular localization of MrSIR2s, we employed a plasmid, pDHt-bar-*gapdh*-GFP, which carries the glyceraldehyde 3-phosphate dehydrogenase (*gapdh*) promoter. This plasmid was utilized for protein fusions, where each MrSIR2 was fused to the terminus of GFP [39]. Subsequently, a DAPI staining assay was conducted to facilitate fluorescence observation.

For the evaluation of autophagic levels and the localization of MrATG8 in different strains, we constructed the plasmid pDHt-ben-*Np*-GFP-MrATG8, which incorporates the native promoter of *Mratg8* [4]. The localization assays were observed through LSCM under various culture conditions.

### Western blotting analysis

Total protein extraction was carried out using a RIPA lysis buffer (Beyotime, P0013K), and the total protein concentration was estimated using the bicinchoninic acid protein assay kit (Beyotime, P0010). In the context of western blotting, 20 or 30 μg of total proteins were loaded and adjusted. The subsequent western blotting analysis utilized anti-GFP antibody (Abmart, M20004), anti-FLAG antibody (Sigma, F1804). To serve as loading controls, parallel-running SDS-PAGE gels were stained and imaged. After 1.5 d, 2.5 d culture on PDA or SDAY, proteins from WT, Δ*Mrsir2–3* or Δ*Mrkat1* strains were immunoprecipitated using anti-FLAG antibody and immunoblotted with pan anti-acetyl-lysine antibody (PTMBiolabs, Hangzhou, China) [21].

### Co-IP assays

The *Rp27* promoter was incorporated into the pDHt-*gapdh*-bar plasmid and utilized to generate the plasmids pDHt-*gapdh*-MrSIR2–3-GFP-bar-*Rp27*-FLAG-MrATG4 and pDHt-*gapdh*-MrKAT1-GFP-bar-*Rp27*-FLAG-MrATG4. The plasmids pDHt-ben-*Rp27*-FLAG, pDHt-*gapdh*-GFP-bar-*Rp27*-FLAG-MrATG4 were constructed and subsequently transformed into the WT or mutant strains to serve as controls. These plasmids were separately introduced into the WT using ATMT. Similarly, point-mutant plasmids, including pDHt-*gapdh*-MrSIR2–3-GFP-bar-*Rp27*-FLAG-MrATG4^*K69R*^, pDHt-*gapdh*-MrSIR2–3-GFP-bar-*Rp27*-FLAG-MrATG4^*K77R*^, pDHt-*gapdh*-MrSIR2–3-GFP-bar-*Rp27*-FLAG-MrATG4^*K149R*^ and pDHt-*gapdh*-MrSIR2–3-GFP-bar-*Rp27*-FLAG-MrATG4^*K69,77R*^, were constructed to examine ATG4 acetylation levels from different point mutant strains.

Total protein was extracted from the transformed strains using a RIPA lysis buffer (Beyotime, P0013K) and subjected to incubation with anti-FLAG Magnetic Beads (Bimake, Shanghai, China) at 4 °C for 10 hours. Immunoblotting was performed using FLAG, GFP or acetyl-lysine antibodies to detect the respective proteins.

### Yeast two-hybrid assays

To investigate potential interactions between MrSIR2–3 and ATG8-conjugation system proteins, or between MrATG4 and various acetyltransferase proteins, yeast two-hybrid assays were performed [4]. Specifically, the plasmids pGBKT7-MrSIR2–3 and pGADT7-MrATG3/4/7/8 were constructed and co-transformed into the yeast strain Y2HGold. Additionally, pGADT7-MrATG4 was co-transformed with pGBKT7-Gcn5/Esa1/Kat1/Rtt109/Sas2/Sas3 into Y2HGold, respectively. As controls, the pair of plasmids pGADT7-T and PGBKT7–53 were used as a positive control, while the paired vectors (pGADT7 and pGBKT7-MrSIR2–3; pGADT7-MrATG4 and pGBKT7) served as negative controls. The interactions were assessed using synthetic dropout-Leu-Trp medium and Leu-Trp-His-Ade/X-α-gal/AbA plates.

### BiFC assays

The plasmids pDHt-*gapdh*-MrSIR2–3-YVN-bar-*gapdh*-MrATG4-YVC and pDHt-*gapdh*-MrKAT1-YVN-bar-*gapdh*-MrATG4-YVC were constructed and then separately transformed into the WT. The plasmids pDHt-*gapdh*-MrSIR2–3-YVN-bar-*gapdh*-YVC, pDHt-*gapdh*-YVN-bar-*gapdh*-MrATG4-YVC, pDHt-*gapdh*-MrKAT1-YVN-bar-*gapdh*-YVC and pDHt-*gapdh*-YVN-bar-*gapdh*-MrATG4-YVC were constructed and then separately transformed into the WT, as the control. The YFP fluorescence was observed under a LSCM [43].

### TEM analysis

Transmission electron microscopy (TEM) was employed to examine the autophagic bodies within the conidia of different strains. The initial conidium production from both the WT and mutant strains were collected, washed, and fixed in 2.5% glutaraldehyde in 0.1 M PBS (pH 7.4) at 4°C for 16 hours. Following three washes with PBS buffer, the samples were dehydrated and embedded in resin. Subsequently, ultrathin sections of each sample were visualized using a TEM (HT-7700, Hitachi, Japan) operating at 80 kV [4,44].

### ATG4 activity assay

ATG4 activity was measured as described previously [45] using the fluorogenic substrate AU4S. Briefly, cells or tissues were lysed in buffer containing 2 mM dithiothreitol (DTT). Lysates were incubated with 0.2 µM AU4S for 40 min at 37°C. Fluorescence intensity was quantified using a luminescence microplate reader (Synergy HTX, BioTek, America).

### Statistical analysis

All data presented in this paper are derived from a minimum of three biological replicates. The two-tailed Student's t-test was employed to assess statistical differences between the WT and various mutants [46]. Significance levels are indicated in the Fig legends, with '*' representing *p* < 0.01.

## Supporting information

S1 Fig Schematic diagram of targeted disruption by homologous recombination approach. (TIF)

S2 Fig Verification of gene deletions. (A) PCR verification of *Mrsir2–1* gene deletion. (B). PCR verification of *Mrsir2–2* gene deletion. (C) PCR verification of *Mrsir2–3* gene deletion. (D) PCR verification of *Mrsir2–4* gene deletion. Within panels A - D, Δ represents the knockout mutant; WT, the wild-type strain; P, the plasmid containing the gene knockout cassette; R, the randomly insert mutants; M, the DNA marker.
(TIF)

S3 Fig Quantification of conidial yields. (A) Quantification of conidial yields from different strains after one weeks of growth on PDA. * *p* < 0.01. (B and C) The conidia production of different strains cultured on CM medium for 7 or 14 days. * *p* < 0.01.
(TIF)

S4 Fig Relative expression levels of conidiation-related genes from different strains. Mrgpdah was used as a control.
(TIF)

S5 Fig BiFC assay for the patterns of MrATG4-MrSIR2-3 and MrATG4-MrKAT1 in *vivo.* (A-D) All the hypha tips were examined by DIC and fluorescence microscopy. Strains expressing MrATG4-nYFP and empty cYFP, MrSIR2–3-cYFP and empty nYFP, MrKAT1-cYFP and empty nYFP were used as negative controls. Scale bar: 10 μm.
(TIF)

S6 Fig Co-immunoprecipitation assays for the patterns of MrATG4 in *vivo.* (A and B) Total proteins, suspensions, and proteins eluted from anti-FLAG agarose from transformants co-expressing Mr-GFP and MrATG4-FLAG. The blots were probed with anti-FLAG or anti-GFP antibodies. T, total; S, suspensions; E, elution.
(TIF)

S7 Fig The fluorescent intensity of GFP-Atg8 in the ∆*Mrsir2–3* mutant and WT. (A-B) The laser power of the confocal microscope was set to a range of 5% to 35% to detect the autophagic fluorescence intensity of ATG8-GFP in WT and ∆*Mrsir2–3* mutant strains. Scale bar: 10 μm.
(TIF)

S8 Fig The fluorescence of various mutants expressing GFP-ATG8. (TIF)

S9 Fig Bioinformatics analysis and verification of gene deletion of MrKAT1. (A) Schematic representation of KAT1 proteins. Kat1_N, histone acetyl transferase KAT1 N-terminus. (B) Phylogenetic analysis of KAT1-related proteins from several fungi. (C) PCR verification of *Mrkat1* gene deletion. Δ represents the knockout mutant; P, the plasmid containing the gene knockout cassette; R, the randomly insert mutants; M, the DNA marker.
(TIF)

S10 Fig Loading control of ATG4 acetylation assay. (A and B) The Mr-FLAG was constructed into WT, Δ*Mrsir2–3* and Δ*Mrkat1* strains. These strains were cultured on PDA or SDAY for 1.5 d, 2.5 d and proteins were immunoprecipitated with antibody to FLAG followed by immunoblotting with antibody to acetylated-lysine.
(TIF)

S11 Fig For the analysis of GFP-MrAtg8 degradation in the WT and mutant mycelial cells cultured in different medium (A and B) The parallel-running protein gels were stained as references, respectively.
(TIF)

S12 Fig Δ*Mratg4* consisted with Δ*Mratg4*^*K69,77,149R*^. (A) Colony phenotyping of WT and mutant strains on different media after growth for 14 days at 25 °C. (B) Quantification of conidial yields for those strains. * *p* < 0.01.
(TIF)

S1 Table Interacting proteins screening of MrSIR2–3. (XLSX)

S2 Table PCR primers used in this study. (XLSX)
