# Supplementary figures and images for: Acetylation dynamics of MrATG4 governing autophagy-mediated conidiation in an entomopathogenic fungus
Source: PLoS Pathog. 2026 Jan 20;22(1):e1013883. doi: 10.1371/journal.ppat.1013883 (PMC12858055; doi:10.1371/journal.ppat.1013883)

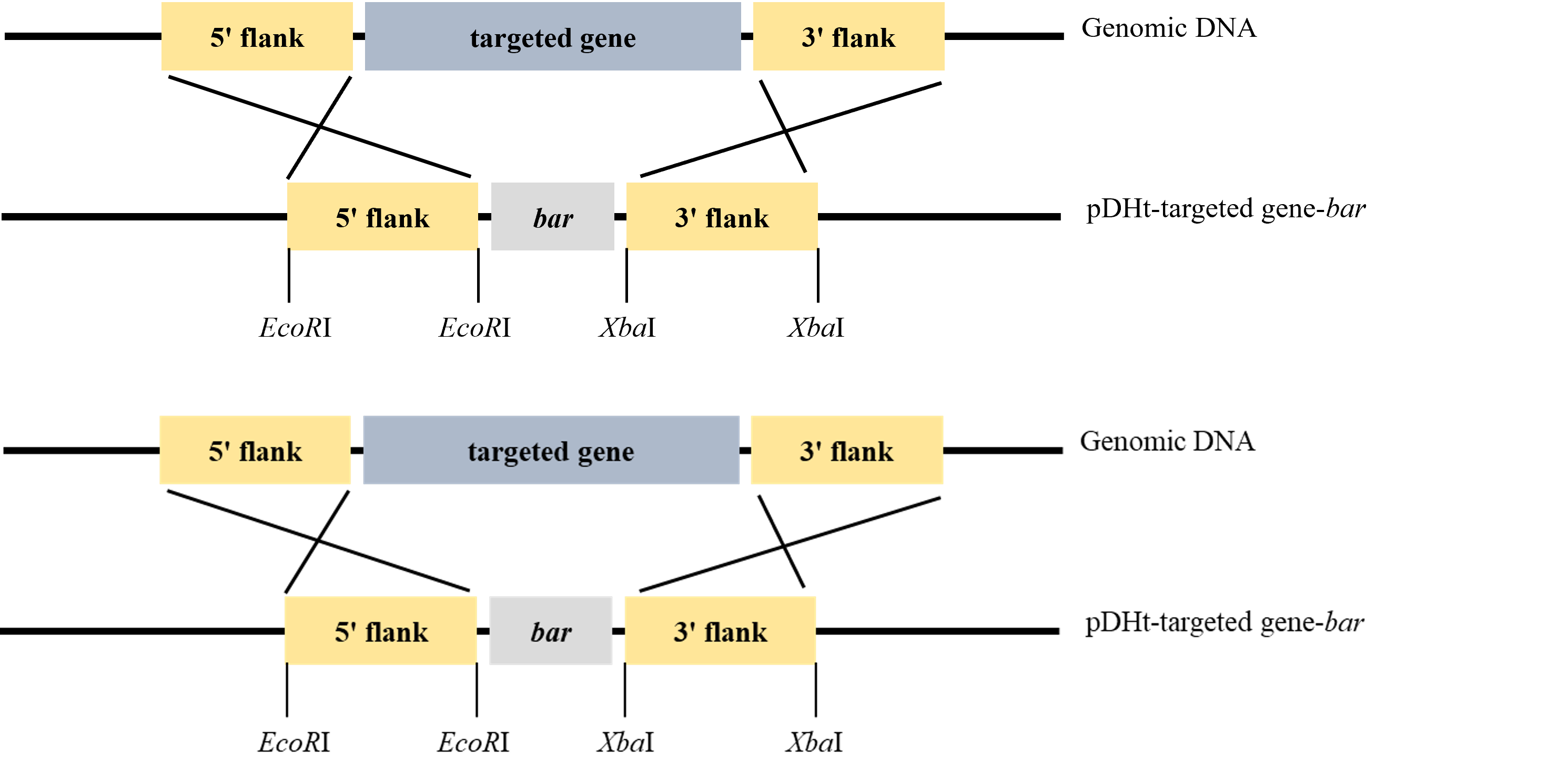

Supplement: S1 Fig — (TIF) [file ppat.1013883.s001.tif]

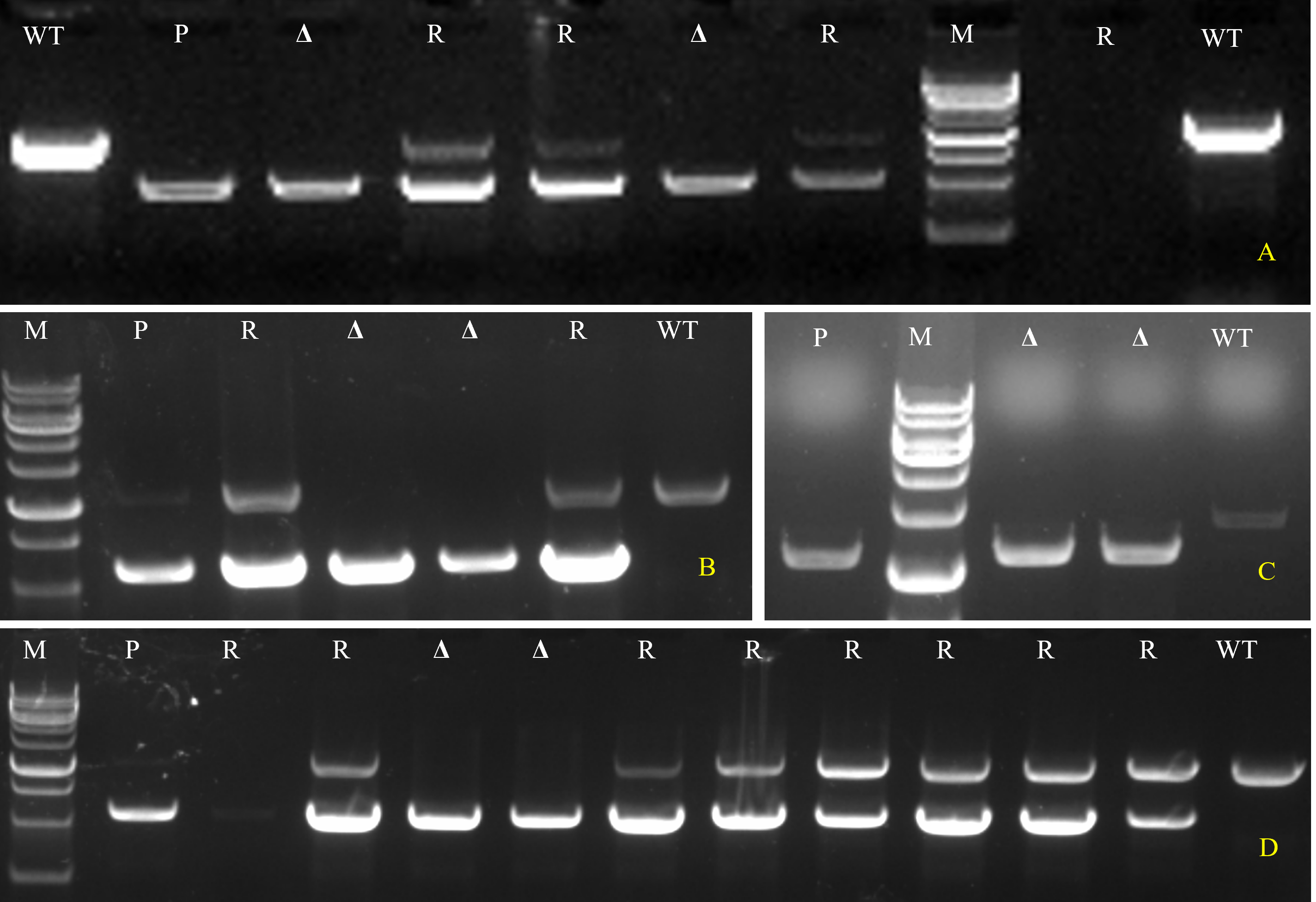

Supplement: S2 Fig — (A) PCR verification of Mrsir2–1 gene deletion. (B). PCR verification of Mrsir2–2 gene deletion. (C) PCR verification of Mrsir2–3 gene deletion. (D) PCR verification of Mrsir2–4 gene deletion. Within panels A - D, Δ represents the knockout mutant; WT, the wild-type strain; P, the plasmid containing the gene knockout cassette; R, the randomly insert mutants; M, the DNA marker. (TIF) [file ppat.1013883.s002.tif]

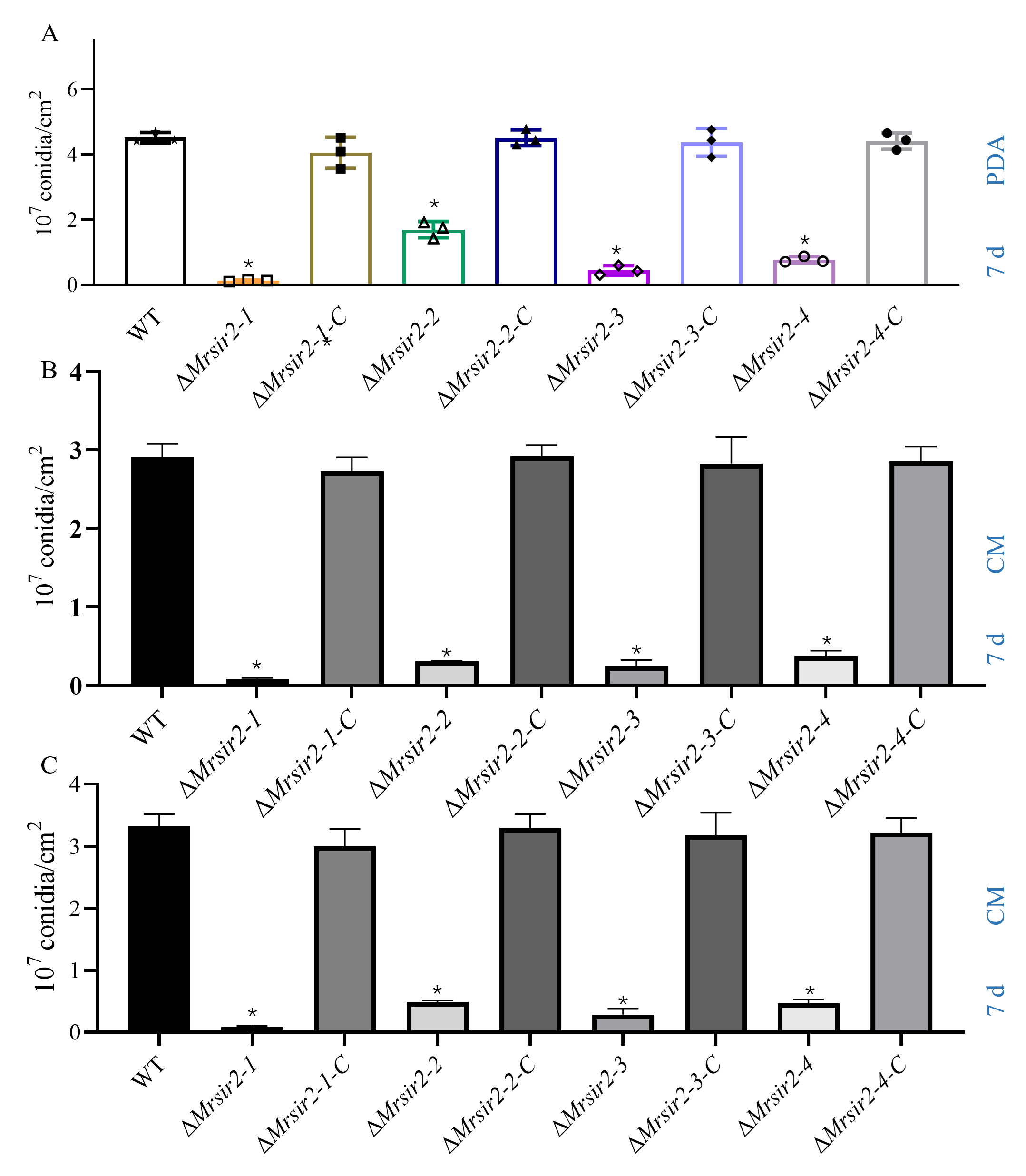

Supplement: S3 Fig — (A) Quantification of conidial yields from different strains after one weeks of growth on PDA. * p < 0.01. (B and C) The conidia production of different strains cultured on CM medium for 7 or 14 days. * p < 0.01. (TIF) [file ppat.1013883.s003.tif]

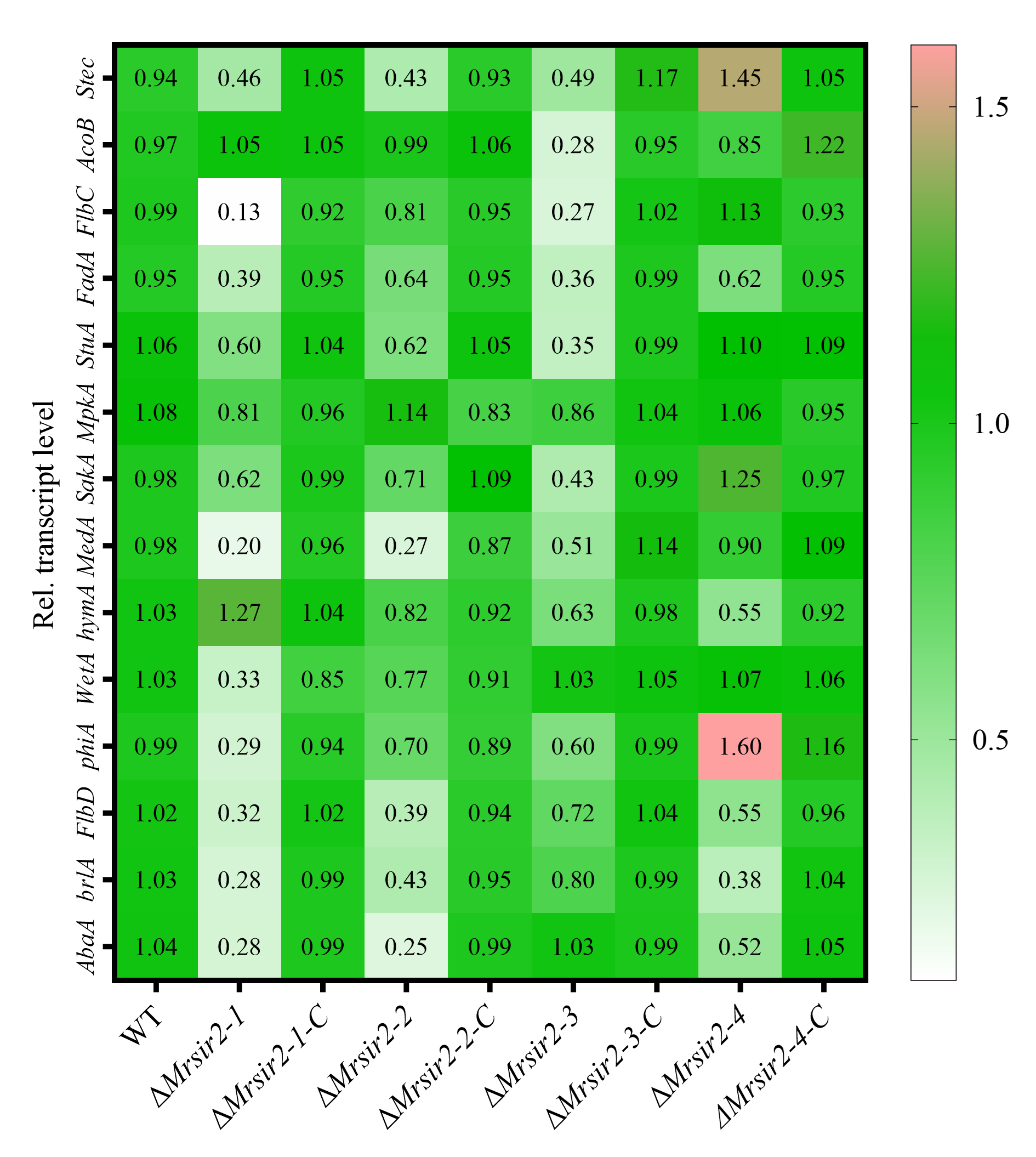

Supplement: S4 Fig — Mrgpdah was used as a control. (TIF) [file ppat.1013883.s004.tif]

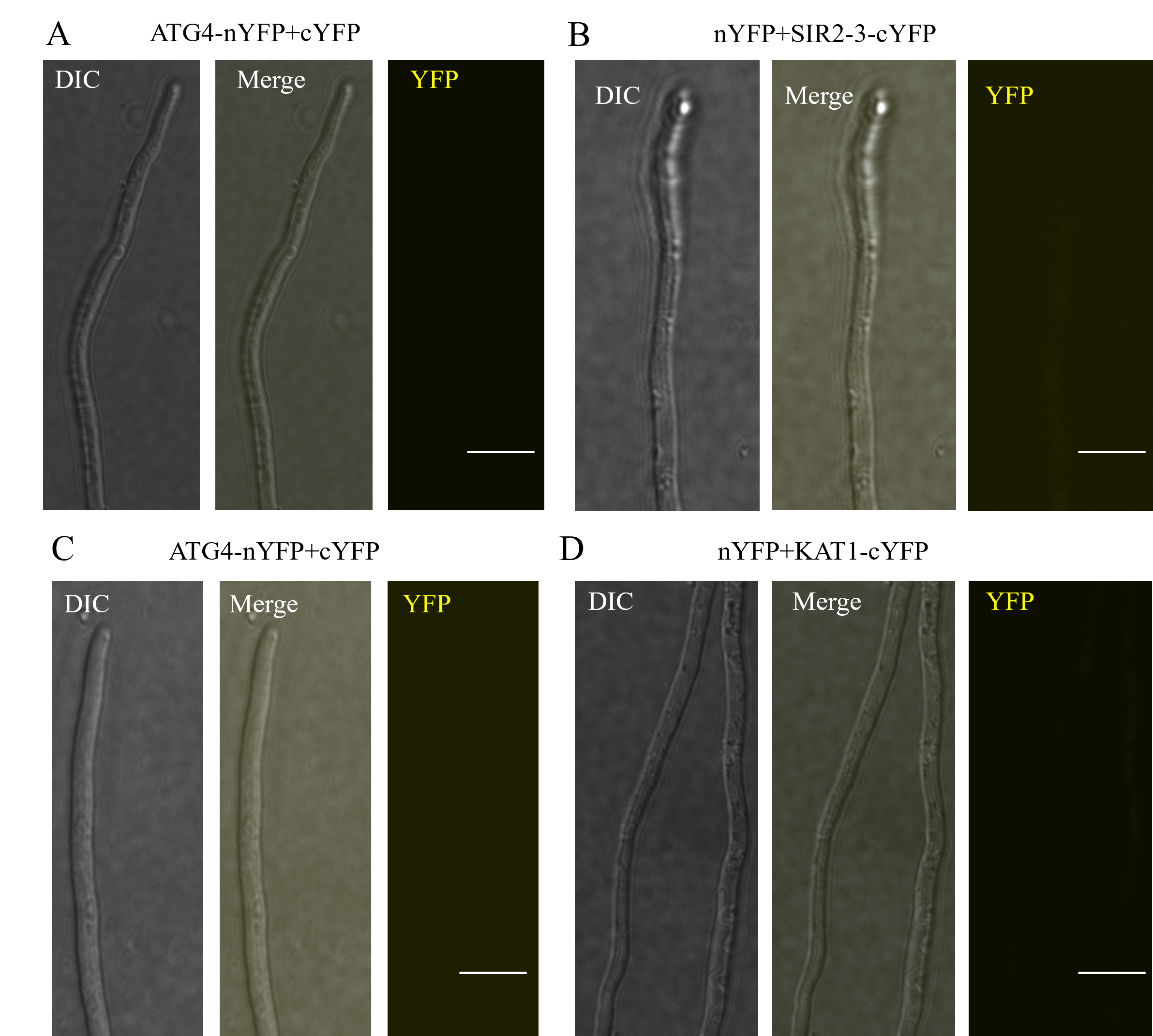

Supplement: S5 Fig — (A-D) All the hypha tips were examined by DIC and fluorescence microscopy. Strains expressing MrATG4-nYFP and empty cYFP, MrSIR2–3-cYFP and empty nYFP, MrKAT1-cYFP and empty nYFP were used as negative controls. Scale bar: 10 μm. (TIF) [file ppat.1013883.s005.tif]

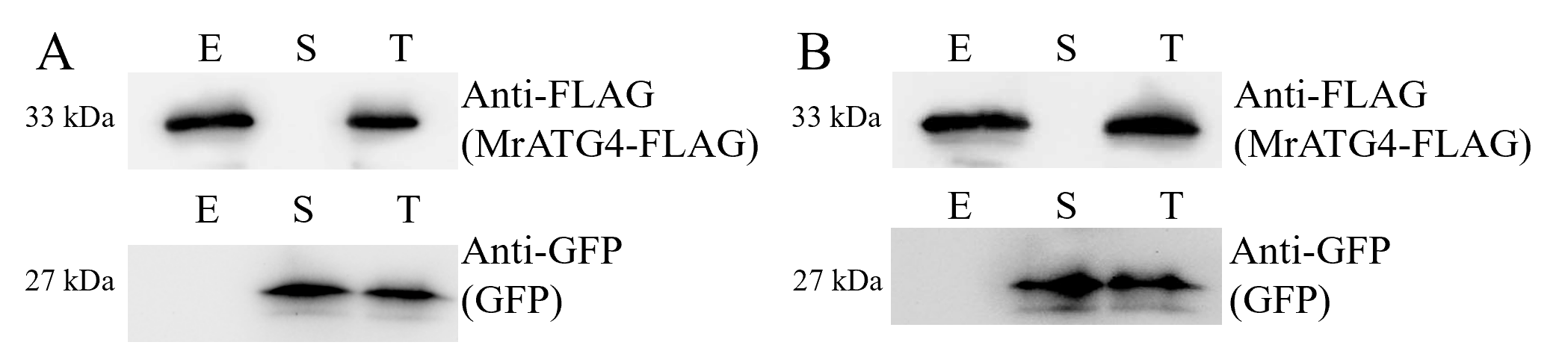

Supplement: S6 Fig — (A and B) Total proteins, suspensions, and proteins eluted from anti-FLAG agarose from transformants co-expressing Mr-GFP and MrATG4-FLAG. The blots were probed with anti-FLAG or anti-GFP antibodies. T, total; S, suspensions; E, elution. (TIF) [file ppat.1013883.s006.tif]

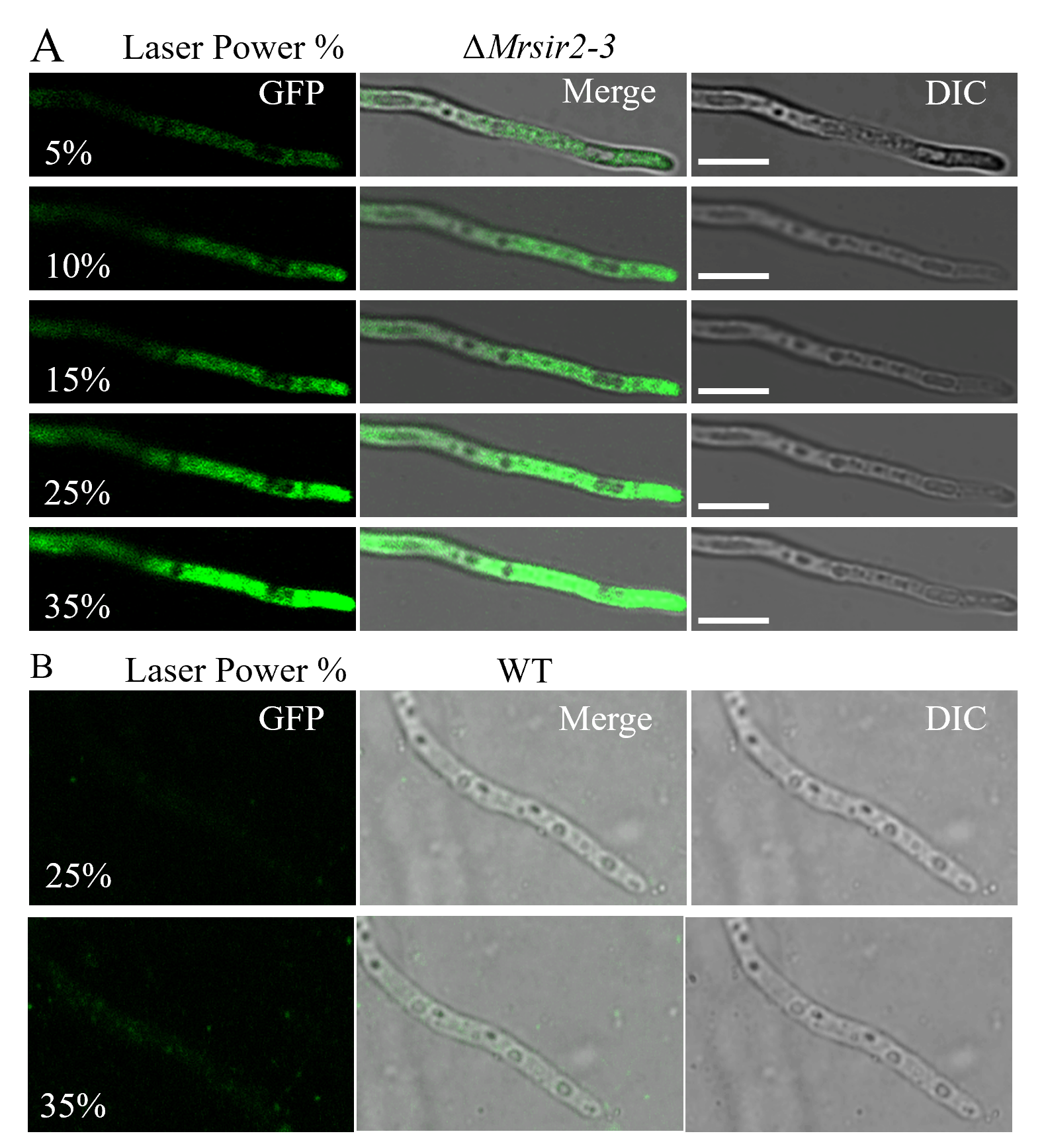

Supplement: S7 Fig — (A-B) The laser power of the confocal microscope was set to a range of 5% to 35% to detect the autophagic fluorescence intensity of ATG8-GFP in WT and ∆Mrsir2–3 mutant strains. Scale bar: 10 μm. (TIF) [file ppat.1013883.s007.tif]

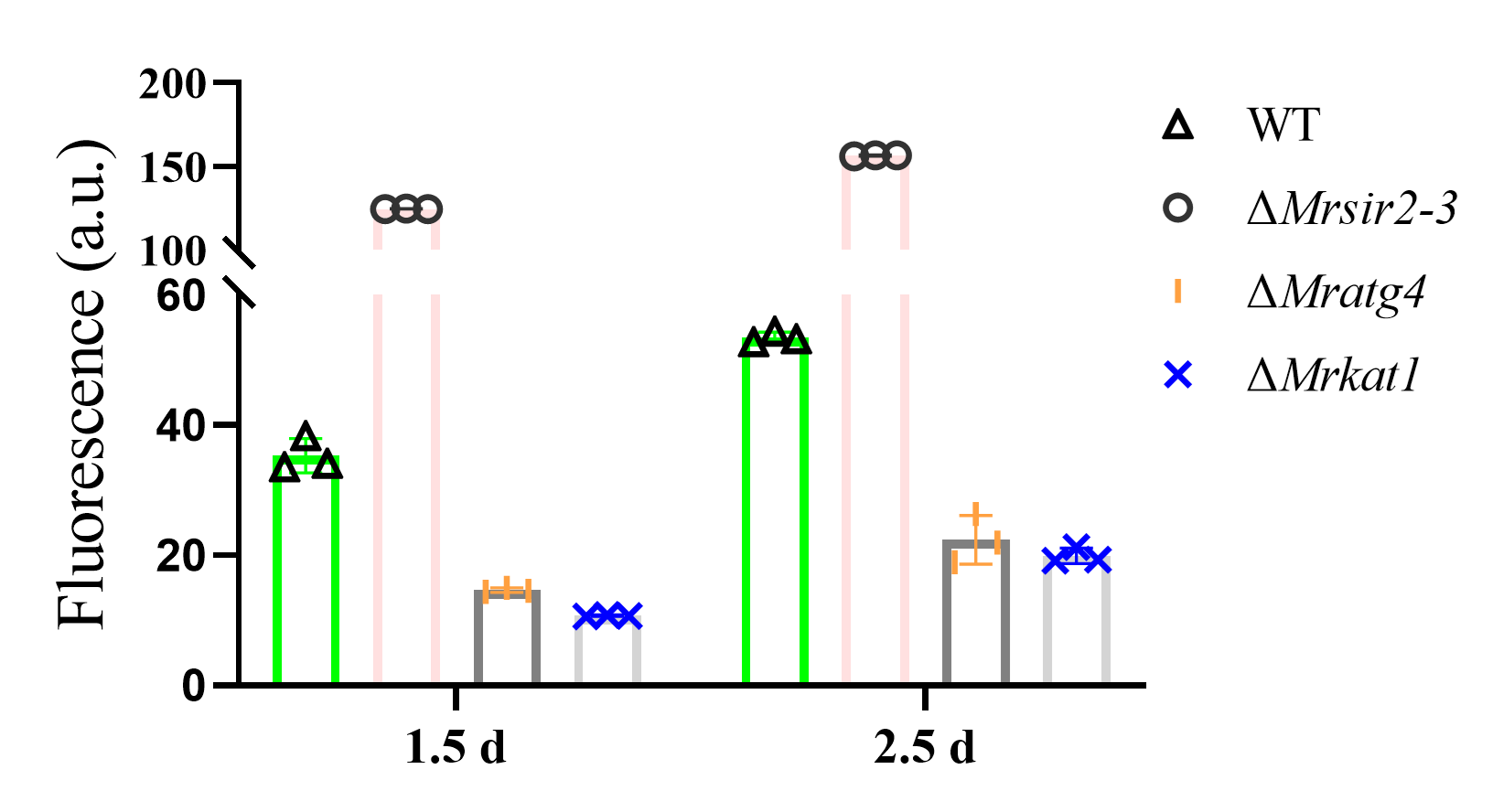

Supplement: S8 Fig — (TIF) [file ppat.1013883.s008.tif]

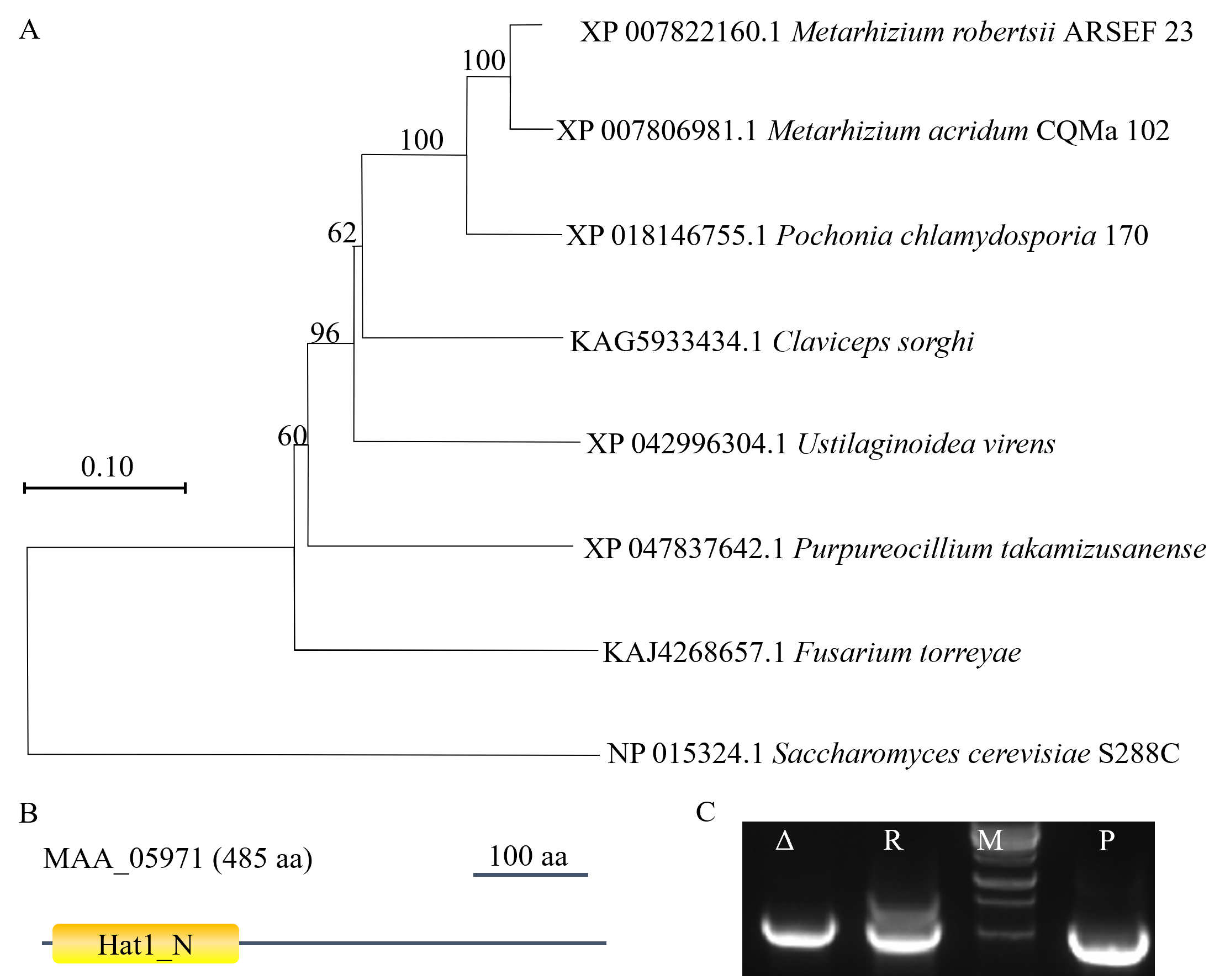

Supplement: S9 Fig — (A) Schematic representation of KAT1 proteins. Kat1_N, histone acetyl transferase KAT1 N-terminus. (B) Phylogenetic analysis of KAT1-related proteins from several fungi. (C) PCR verification of Mrkat1 gene deletion. Δ represents the knockout mutant; P, the plasmid containing the gene knockout cassette; R, the randomly insert mutants; M, the DNA marker. (TIF) [file ppat.1013883.s009.tif]

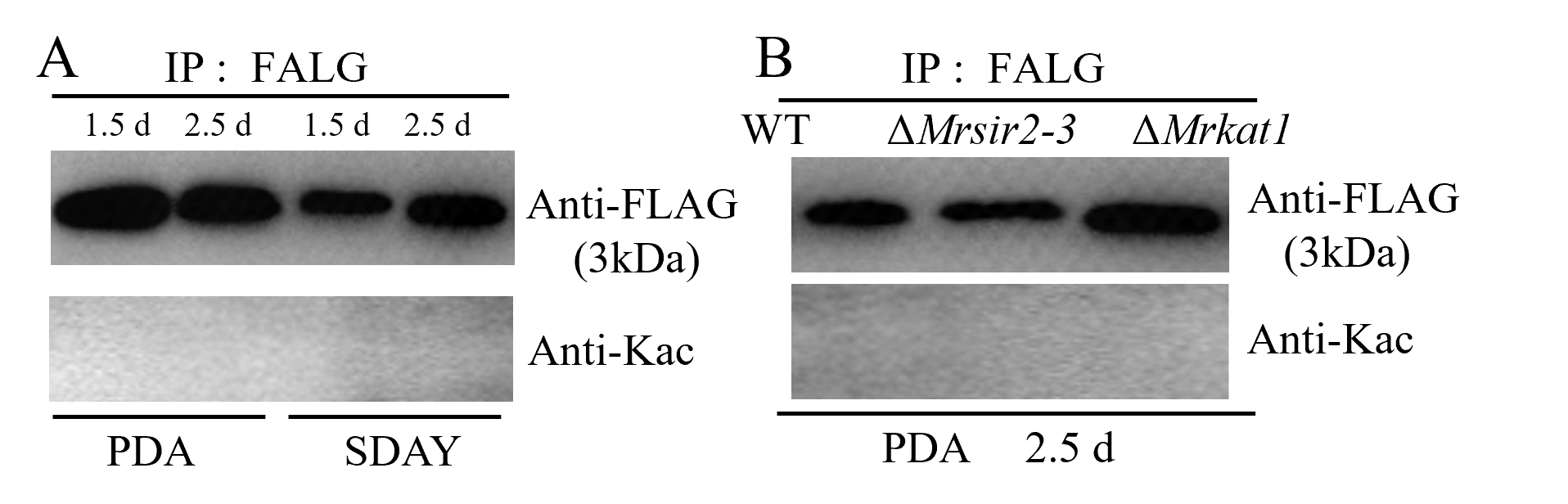

Supplement: S10 Fig — (A and B) The Mr-FLAG was constructed into WT, ΔMrsir2–3 and ΔMrkat1 strains. These strains were cultured on PDA or SDAY for 1.5 d, 2.5 d and proteins were immunoprecipitated with antibody to FLAG followed by immunoblotting with antibody to acetylated-lysine. (TIF) [file ppat.1013883.s010.tif]

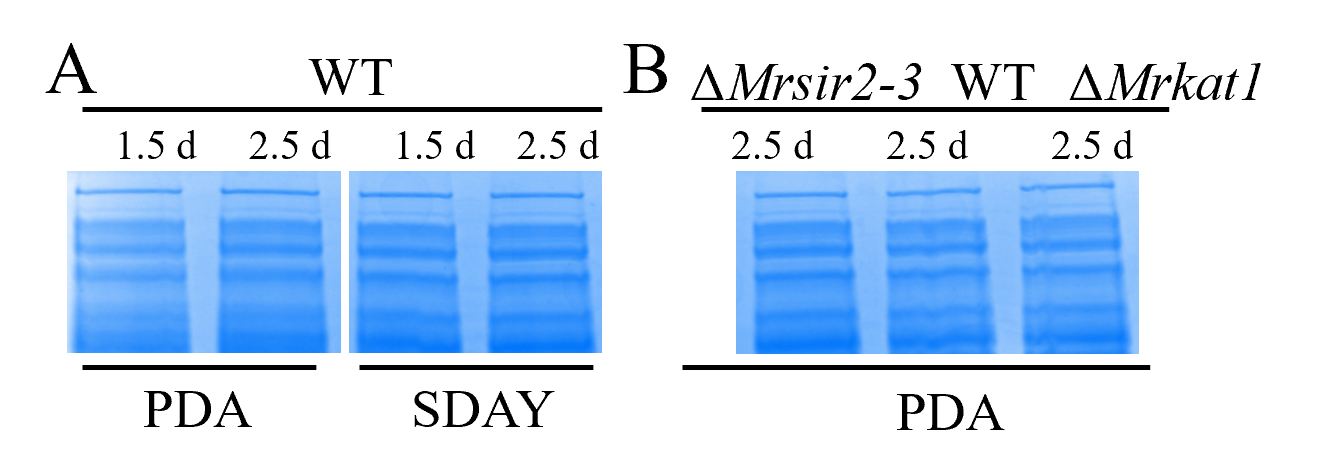

Supplement: S11 Fig — (A and B) The parallel-running protein gels were stained as references, respectively. (TIF) [file ppat.1013883.s011.tif]

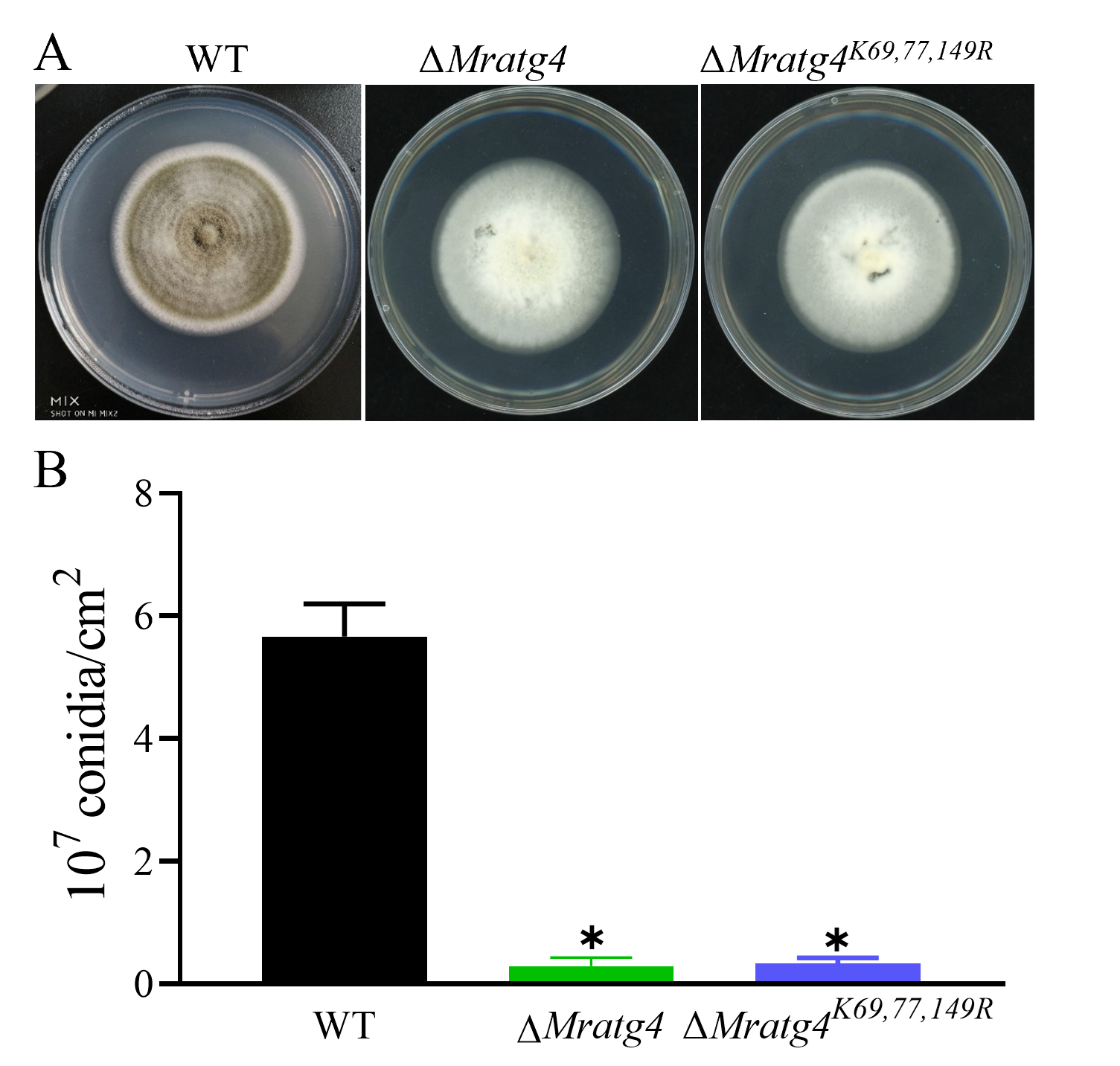

Supplement: S12 Fig — (A) Colony phenotyping of WT and mutant strains on different media after growth for 14 days at 25 °C. (B) Quantification of conidial yields for those strains. * p < 0.01. (TIF) [file ppat.1013883.s012.tif]
